# Cost-effectiveness analysis of timely dialysis referral after renal transplant failure in Spain

**DOI:** 10.1186/1472-6963-12-257

**Published:** 2012-08-16

**Authors:** Guillermo Villa, Emilio Sánchez-Álvarez, Jesús Cuervo, Lucía Fernández-Ortiz, Pablo Rebollo, Francisco Ortega

**Affiliations:** 1 BAP LA-SER Outcomes, LA-SER Group, Azcárraga, 12 A, 33010, Oviedo, Asturias, Spain; 2 Department of Medicine, Universidad de Oviedo, Oviedo, Spain; 3 Department of Nephrology, Hospital Universitario Central de Asturias, Oviedo, Spain

**Keywords:** Chronic kidney disease, Cost-effectiveness analysis, Timely dialysis referral, Graft function loss, Kidney transplantation, Late dialysis referral, Markov models, Renal replacement therapy, Transplant failure

## Abstract

**Background:**

A cost-effectiveness analysis of timely dialysis referral after renal transplant failure was undertaken from the perspective of the Public Administration. The current Spanish situation, where all the patients undergoing graft function loss are referred back to dialysis in a late manner, was compared to an ideal scenario where all the patients are timely referred.

**Methods:**

A Markov model was developed in which six health states were defined: hemodialysis, peritoneal dialysis, kidney transplantation, late referral hemodialysis, late referral peritoneal dialysis and death. The model carried out a simulation of the progression of renal disease for a hypothetical cohort of 1,000 patients aged 40, who were observed in a lifetime temporal horizon of 45 years. In depth sensitivity analyses were performed in order to ensure the robustness of the results obtained.

**Results:**

Considering a discount rate of 3 %, timely referral showed an incremental cost of 211 €, compared to late referral. This cost increase was however a consequence of the incremental survival observed. The incremental effectiveness was 0.0087 quality-adjusted life years (QALY). When comparing both scenarios, an incremental cost-effectiveness ratio of 24,390 €/QALY was obtained, meaning that timely dialysis referral might be an efficient alternative if a willingness-to-pay threshold of 45,000 €/QALY is considered. This result proved to be independent of the proportion of late referral patients observed. The acceptance probability of timely referral was 61.90 %, while late referral was acceptable in 38.10 % of the simulations. If we however restrict the analysis to those situations not involving any loss of effectiveness, the acceptance probability of timely referral was 70.10 %, increasing twofold that of late referral (29.90 %).

**Conclusions:**

Timely dialysis referral after graft function loss might be an efficient alternative in Spain, improving both patients’ survival rates and health-related quality of life at an affordable cost. Spanish Public Health authorities might therefore promote the inclusion of specific recommendations for this group of patients within the existing clinical guidelines.

## Background

Kidney transplantation is the treatment of choice for Chronic Kidney Disease (CKD) [1]. In the last decades, the progressive improvement of immunosuppressant drugs has led to an increase in the survival of renal grafts. It has been shown that the one-year survival rate of a renal graft is above 90%. The five-year survival rate is nevertheless around 70% and survival after ten years is below 50% [2]. Every year, therefore, many patients experience graft function loss, being referred back to dialysis. In a recent study carried out by Villa et al., it is estimated that around 4% of the Spanish patients in kidney transplantation would be referred back to dialysis every year, adding up to almost 1,000 patients in 2010 [3].

Despite the existence of a number of clinical practice guidelines, both at the national and the international levels, there is no consensus on the right timing for dialysis referral after graft function loss. Both the reticence of clinicians to assume transplant failure and the reluctance of patients to restart dialysis might be among the causes of late dialysis referral. Moreover, kidney transplantation management and research have traditionally focused on immunosuppressant therapy and on the management of complications, rather than on the condition of patients restarting dialysis.

Following the international recommendations, there are two situations in which patients should start Renal Replacement Therapy (RRT) [4-6]: (1) Glomerular Filtration Rate (GFR) below 15 ml/min/1.73 m^2^ (i.e. Stage 5 of CKD) and presence of uremic complications; and (2) GFR below 6 ml/min/1.73 m^2^, even in the absence of symptoms. In the case of elder patients or in the presence of comorbidities, it is however recommended an early RRT start, even though GFR is above 15 ml/min/1.73 m^2^ and there is absence of symptoms. Recent studies propose however that dialysis initiation is justified at GFR levels from 5 to 9 ml/min/1.73 m^2^ if accompanied by symptoms [7].

Arias et al. found that patients experiencing graft function loss presented GFR of 9 ml/min/1.73 m^2^ at the time of hemodialysis restart, with 78 % of the patients showing GFR of less than 10 ml/min/1.73 m^2^[8]. Likewise, Gill et al. found GFR of 8.4 ml/min/1.73 m^2^ for a similar group of patients [9]. In both cases, GFR was below the current recommendations. Late dialysis referral usually involves a non-scheduled (non-programmed, non-planned or urgent) dialysis restart, what has important clinical [10,11] and economic [3,12] implications, such as higher undernourishment, worse anemic control, higher morbidity and mortality rates, and consequently larger costs. Furthermore, patients undergoing graft function loss show higher recombinant human erythropoietin (rHuEPO) [13] and intravenous iron (IV) [14] needs, experience higher hospitalization rates due to access complications [7] and face increased morbidity and mortality risks [8,15-20]. Because of that, a timely dialysis restart would be advisable for these patients as soon as they reach Stage 5 of CKD.

This article studies the health outcomes and the economic implications of late dialysis referral after graft function loss. A cost-effectiveness analysis of timely dialysis referral after renal transplant failure is undertaken for the first time in Spain. The current Spanish situation is compared to an ideal scenario in which all the patients undergoing graft function loss are referred back to dialysis in a timely manner.

## Methods

A Markov chain model was programmed using Stata 10 data analysis software. Markov models are useful to represent random processes which evolve over time. They are suited to modeling the progression of chronic diseases. A specific disease is described as a chain of different health states, and movements between those states over discrete time periods (“cycles”) occur with a given probability (“transition probability”). Estimates of health outcomes and costs are attached to each state in the model. By running the model over a large number of cycles (“temporal horizon”), the long-term health outcomes and costs associated with the disease are computed (see [21] for a detailed introduction to Markov modeling).

In our particular case, six health states were defined: HD: hemodialysis; PD: peritoneal dialysis; Tx: kidney transplantation; LRHD: late referral hemodialysis; LRPD: late referral peritoneal dialysis; and D: death. The model carried out a simulation of the progression of renal disease for a hypothetical cohort of 1,000 patients aged 40, the most frequent age for RRT initiation according to expert judgment, who were observed in a lifetime temporal horizon of 45 years. The model parameters and their supporting references are presented in Table 1.

**Table 1:** Model parameters

	**Global**	**HD**	**PD**	**Tx**	**LRHD**	**LRPD**	**D**	**References**
**Direct medical costs: first-year**		2,545 €	1,819 €	36,772 €	6,627 €	3,748 €	0 €	Villa et al. (2011) [12] and this study
**Direct medical costs: prevalence**		31,912 €	24,996 €	6,030 €	31,912 €	24,996 €	0 €	Villa et al. (2011) [12] and this study
**Health utilities**		0.69	0.69	0.81	0.53	0.53	0.00	Villa et al. (2012) [3] and Laupacis et al. (1996) [25]
**Transition probabilities from/to HD, PD, LRHD, LRPD, Tx**	See Figure 1							Villa et al. (2011) [3]
**Age-dependent transition probabilities to D**								SEN (2008) [22], Kaplan et al. (2002) [23] and this study
**Starting cohort (patients)**	1,000							Arbitrary
**Starting age (years)**	40							Expert panel opinion
**Time horizon (years)**	45							Expert panel opinion
**WTPT (€/QALY)**	45,000							De Cock et al. (2007) [26]
**Discount rate**	3 %							López-Bastida et al. (2010) [27]

HD: hemodialysis.

PD: peritoneal dialysis.

Tx: kidney transplantation.

LRHD: late referral hemodialysis.

LRPD: late referral peritoneal dialysis.

D: death.

WTPT: willingness-to-pay treshold.

QALY: quality-adjusted life year.

**Figure 1:**
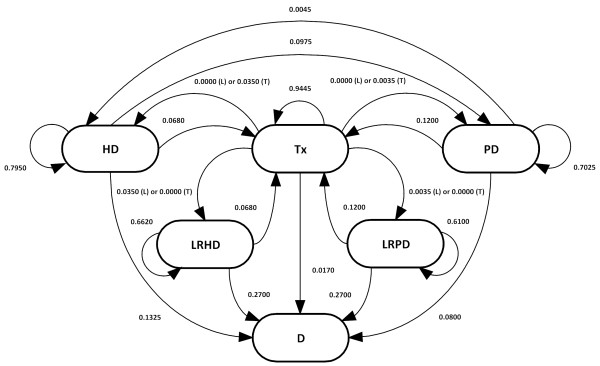
**Transition probabilities.** HD: hemodialysis. PD: peritoneal dialysis. Tx: kidney transplantation. LRHD: late referral hemodialysis. LRPD: late referral peritoneal dialysis. D: death. T: Scenario T. L: Scenario L.

Transition probabilities determine the likelihood of patient flows between the health states defined from cycle (a year) to cycle (Figure 1). Transition probabilities were based on a recent study [3], with the exception of age-dependent mortality probabilities, which were computed using data from the Spanish Society of Nephrology (SEN, Spanish acronym) registry [22] and assuming that late referral patients had a one-year survival rate of 73% [23]. All the model transitions were half-cycle corrected [24].

Based on the opinion of an expert panel of three clinicians, two alternative scenarios were considered:

▪ Scenario L. All the patients are referred back to dialysis (both to HD and PD) in a late manner after graft function loss (transition probabilities: Tx to HD: 0.0000; Tx to PD: 0.0000; Tx to LRHD: 0.0350; Tx to LRPD: 0.0035). Scenario L represents the current Spanish situation, according to the expert panel opinion.

▪ Scenario T. All the patients are referred back to dialysis in a timely manner after graft function loss (transition probabilities: Tx to HD: 0.0350; Tx to PD: 0.0035; Tx to LRHD: 0.0000; Tx to LRPD: 0.0000). Scenario T represents an ideal situation.

Following a Public Administration perspective, direct medical costs (in January 2012 €), including the costs of the vascular (HD) or peritoneal (PD) accesses; access complications; delivery of training; treatment session; kidney transplantation; drug consumption; equipment depreciation and maintenance; Nephrology Service general expenses; utilities; and external services, were considered. HD, PD and Tx transition and prevalence costs were collected from a recent study. Transition (incidence or first-year) costs include the costs of the vascular access (HD), the costs of the peritoneal access and training (PD), and the costs of transplantation surgery (Tx).

LRHD and LRPD costs were based on that same methodology [12], but taking into account some considerations. It was considered that LRHD patients required a higher (Δ97%) number of days of hospitalization due to access complications than HD patients did [7]. It was further considered that, during the first year, LRHD and LRPD patients showed higher (Δ69 %) rHuEPO [13] and higher (Δ47%) IV [14] needs than HD and PD patients did. Moreover, both LRHD and LRPD patients were considered as non-scheduled (i.e. they start dialysis in a non-planned, non-programmed or urgent form). Per-patient annual transition costs were: 2,545 € (HD) < 6,627 € (LRHD) and 1,819 € (PD) < 3,748 € (LRPD).

Effectiveness was expressed in terms of quality-adjusted life years (QALY). QALY were defined as the survival rate in a cycle times the health utility associated with a given health state. Health utilities were assigned values on a scale from 0 (the worst health state or death) to 1 (the optimal or perfect health state). Utilities were obtained from the existing literature [3,25]. Regarding LRHD and LRPD utilities, we considered the average (first year) post-transplant “good dialysis state” utility reported by Laupacis et al. [25]. This study has been cited by a number of relevant studies for similar purposes [28-31]. This estimate presents however some limitations. First, patients (and not the society) were asked to evaluate hypothetical health states using the time trade-off method. Second, health states evaluated were not based on generic quality of life instruments.

Both costs and health utilities were applied a 3% discount rate [27]. As for the cost-effectiveness comparisons, the incremental cost-effectiveness ratio (ICER) was computed and a willingness-to-pay threshold (WTPT) of 45,000 €/QALY [26] was assumed.

For the purpose of contrasting the robustness of the results, univariate and probabilistic (Monte Carlo simulation) sensitivity analyses were carried out. In the univariate case, each single model parameter was changed (a 10% increase or decrease) at a time and a new ICER was computed. In the probabilistic case, 1,000 new ICER were computed by changing all the model parameters simultaneously. Beta distributions were assumed for the transition probabilities, normal distributions were assumed for the health utilities, log-normal distributions were assumed for the costs and a uniform distribution was assumed for the discount rate [32]. Due to data unavailability and when required, standard deviations were assumed to be a 10% of the mean values. The cost-effectiveness plane and the confidence ellipse (95% confidence level) were developed. The probabilities of accepting the two scenarios proposed as a function of the WTPT (acceptability curves) were also computed.

## Results

Cost-effectiveness results are shown in Table 2. Per-patient annual costs and QALY are presented for both scenarios, as well as comparative measures between them.

**Table 2:** Results: Scenario L vs Scenario T

	**Scenario L**	**Scenario T**	**Comparative**
**Deterministic analysis**			
Per-patient annual cost	5,793 €	6,217 €	425 €
Per-patient annual cost (discount rate 3 %)	4,564 €	4,775 €	211 €
Per-patient annual QALY	0.2250	0.243	0.0176
Per-patient annual QALY (discount rate 3 %)	0.1594	0.1682	0.0087
ICER			24,135 €
ICER (discount rate: 3 %)			24,390 €
**Probabilistic analysis (3 % discount rate)**			
Per-patient annual cost (95 % confidence interval)	4,591 € (3,926 €; 5,422 €)	4,771 € (4,073 €; 5,630 €)	180 € (−898 €; 1,305 €)
Per-patient annual QALY (95 % confidence interval)	0.1594 (0.1372; 0.1815)	0.1682 (0.1446; 0.1947)	0.0088 (−0.0245; 0.0431)
Dominant	20.20 %	27.80 %	7.60 %
Efficient (higher effectiveness)	3.20 %	27.60 %	24.40 %
Efficient (lower cost)	14.70 %	6.50 %	−8.20 %
Acceptable	38.10 %	61.90 %	23.80 %
Acceptable (without loss of effectiveness)	29.90 %	70.10 %	40.20 %

ICER: incremental cost-effectiveness ratio.

Considering a discount rate of 3%, Scenario T showed an incremental average (per-patient and year) cost of 211 €, compared to Scenario L. This average cost increase was however due to the incremental average survival observed in Scenario T. The incremental effectiveness was 0.0087 QALY. When comparing both scenarios, an ICER of 24,390 €/QALY was obtained, meaning that Scenario T is an efficient alternative if we consider a WTPT of 45,000 €/QALY.

**Figure 2:**
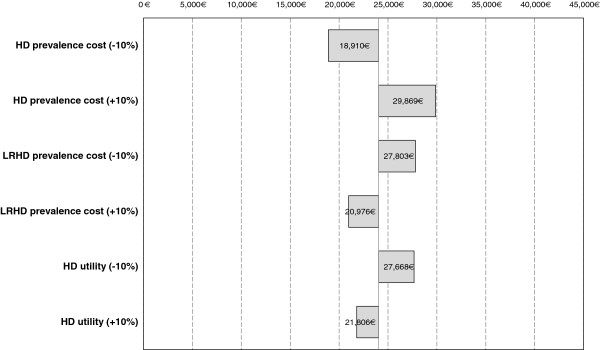
**Incremental cost-effectiveness ratio for univariate parameter changes (Tornado).** HD: hemodialysis. LRHD: late referral hemodialysis.

The univariate sensitivity analysis showed that model results were robust. The ICER did not change significantly when alternative discount rates of 0% (24,135 €/QALY) or 5% (24,405 €/QALY) were considered. Only three model parameters caused absolute value changes in the ICER exceeding a 10% threshold: HD and LRHD prevalence costs, and HD utilities (Figure 2). A maximum ICER of 29,869 €/QALY, yet below the WTPT, was obtained by increasing the prevalence cost of HD in a 10%. Finally, a 10% increase in the utility of LRHD patients caused a 6.01% increase in the ICER, meaning that the influence of this parameter on the results is limited.

In this study, two extreme situations were considered in which either all the patients were referred back to dialysis in a late manner (Scenario L) or all of them were timely referred (Scenario T). A univariate sensitivity analysis was conducted on the proportion of patients who were referred back to dialysis in a late manner. As expected, it was concluded that the higher the proportion of late referral patients considered, the lower the ICER obtained. The ICER was furthermore below the WTPT for any proportion of late referral patients, ranging from 24,428 €/QALY (proportion of late referral patients equal to 0.01) to 24,390 €/QALY (Scenario L). Timely dialysis referral was therefore an efficient alternative for any proportion of late referral patients observed.

**Figure 3:**
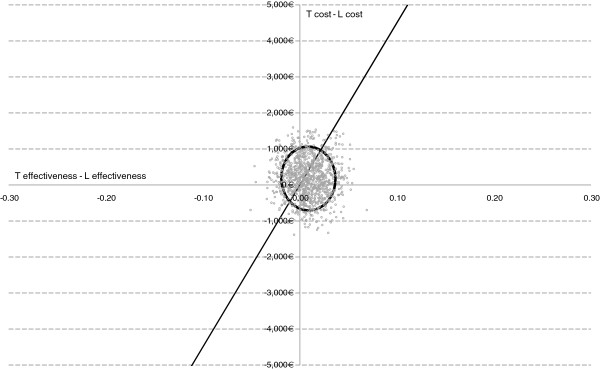
**Cost-effectiveness plane and 95 % confidence ellipse.** T: Scenario T. L: Scenario L.

**Figure 4:**
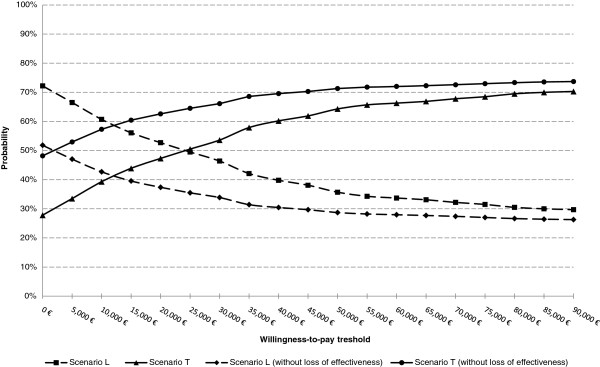
Acceptability curves.

The probabilistic sensitivity analysis (Table 2, Figure 3) showed that, given a WTPT of 45,000 €/QALY, Scenario T was a dominant alternative in 27.80%, efficient with higher effectiveness in 27.60% and efficient with lower costs in 6.50% of the simulations. In contrast, Scenario L was a dominant alternative in 20.20%, efficient with lower cost in 14.70% and efficient with higher effectiveness in 3.20% of the simulations. The acceptance probability of Scenario T was 61.90%, while Scenario L was acceptable in 38.10% of the simulations. If we however restrict the analysis to those situations not involving any loss of effectiveness (i.e. situations of dominance and efficiency with higher effectiveness), the acceptance probability of Scenario T was 70.10%, doubling that of Scenario L (29.90%). Figure 4 shows the acceptability curves of the two scenarios considered for any WTPT ranging from 0 €/QALY to 90,000 €/QALY.

## Discussion

This study presented a cost-effectiveness analysis of timely dialysis referral after renal transplant failure in Spain from the perspective of the Public Administration. Late dialysis referral after graft function loss usually involves a non-scheduled dialysis restart that has important clinical and economic implications, such as higher undernourishment, worse anemic control, higher morbidity and mortality rates, and consequently larger costs incurred. The health outcomes and the economic resources associated with late dialysis referral after graft function loss were discussed and quantified.

A Markov chain model was developed and the current Spanish situation, where the great majority of patients are referred back to dialysis in a late manner, was compared to an ideal scenario in which all the patients undergoing graft function loss were timely referred. In depth sensitivity analyses were performed in order to ensure the robustness of the results obtained.

LRHD and LRPD patients presented lower survival rates and health utilities, and higher transition and prevalence costs than HD and PD patients did. Assuming a WTPT of 45,000 €/QALY, timely dialysis referral might be an efficient alternative when compared to the current Spanish situation. This result proved to be independent of the proportion of late referral patients considered, since the ICER was below the WTPT for any proportion of late referral patients.

Timely dialysis referral implied a moderate increase in total costs. This cost increase was nevertheless caused by the increased survival rates observed in the timely referral scenario, since all the prevalent patients require a lifelong treatment. In real life, the additional costs associated with starting dialysis 6 or 12 months earlier might also contribute to a cost increase. Simulations were re-run only considering those situations not involving any loss of effectiveness. Following this approach, the acceptance probability of timely dialysis referral increased twofold that one of the current Spanish scenario. Based on these results, nephrologists might inform patients on the increased morbidity and mortality risks associated with late dialysis referral.

A limitation of this study is that costs and outcomes in our model are mainly based on single punctual estimates gathered from the existing literature, due to unavailability of micro-data in our country. We were therefore unable to attach confidence intervals to the vast majority of model parameters and had to assume a dispersion of 10% of the central value for all the model parameters. We suspect that the ICER dispersion might be overestimated in our model and therefore the results of the probabilistic sensitivity analysis should be taken with caution.

It is worth noting that late referral patients are also expected to incur higher loss of labor productivity costs due to morbidity and mortality than timely referral patients do. A second limitation of this study is that a Public Administration perspective was adopted rather than including indirect costs (societal perspective), since reliable estimates of the unemployment and retirement rates for late referral patients are not available in Spain and further research would be required. In an exploratory analysis, we quantified loss of labor productivity costs due to mortality in 29,345 € per death and year for RRT patients under 67 years old [12,33], resulting in an ICER of −146 €/QALY when included in the model. The inclusion of loss of labor productivity costs due to morbidity is expected to reduce the ICER obtained, further validating the timely referral approach proposed.

## Conclusions

Timely dialysis referral after graft function loss might be an efficient alternative in Spain, improving both patients’ survival rates and health-related quality of life at an affordable cost. Spanish Public Health authorities might promote the inclusion of specific recommendations for this group of patients within the existing clinical guidelines, also monitoring their proper implementation and outcomes.

## Abbreviations

CKD = Chronic kidney disease; D = Death; GFR = Glomerular filtration rate; HD = Hemodialysis; ICER = Incremental cost-effectiveness ratio; IV = Intravenous iron; LRHD = Late referral hemodialysis; LRPD = Late referral peritoneal dialysis; PD = Peritoneal dialysis; QALY = Quality-adjusted life year; rHuEPO = recombinant human erythropoietin; RRT = Renal replacement therapy; SEN (Spanish acronym) = Sociedad Española de Nefrología; Tx = Kidney transplantation; WTPT = Willingness-to-pay threshold.

## Competing interests

The authors declare that they have no competing interests.

## Authors’ contributions

GV has contributed to the conception and design of the study. He has managed the acquisition, analysis and interpretation of data, and has been also involved in drafting the manuscript. JC and LFO have participated in the design of the study and in the acquisition and interpretation of data. They have been also involved in critically revising the manuscript. ESA, PR, FO have contributed to the conception of the study and have participated in the interpretation of data. They have been also involved in critically revising the manuscript. Finally, all the authors have given final approval of the version to be published.

## Pre-publication history

The pre-publication history for this paper can be accessed here:

http://www.biomedcentral.com/1472-6963/12/257/prepub
